# Selective Constraint on Noncoding Regions of Hominid Genomes

**DOI:** 10.1371/journal.pcbi.0010073

**Published:** 2005-12-16

**Authors:** Eliot C Bush, Bruce T Lahn

**Affiliations:** Howard Hughes Medical Institute, Department of Human Genetics, University of Chicago, Chicago, Illinois, United States of America; University of California Santa Cruz, United States of America

## Abstract

An important challenge for human evolutionary biology is to understand the genetic basis of human–chimpanzee differences. One influential idea holds that such differences depend, to a large extent, on adaptive changes in gene expression. An important step in assessing this hypothesis involves gaining a better understanding of selective constraint on noncoding regions of hominid genomes. In noncoding sequence, functional elements are frequently small and can be separated by large nonfunctional regions. For this reason, constraint in hominid genomes is likely to be patchy. Here we use conservation in more distantly related mammals and amniotes as a way of identifying small sequence windows that are likely to be functional. We find that putatively functional noncoding elements defined in this manner are subject to significant selective constraint in hominids.

## Synopsis

A major goal of human evolutionary biology is to understand what genetic changes make humans unique. One influential idea is that changes in gene expression are most responsible for unique human characteristics. Regulatory elements in noncoding DNA play a key role in controlling gene expression, so one approach is to study human–chimpanzee differences in these elements. Here we use conservation in more distantly related mammals and amniotes as a way of identifying small sequence windows that are likely to be functional. We find that putatively functional noncoding elements defined in this manner are subject to significant selective constraint in hominids. Contrary to some previous reports, these results argue that hominid noncoding regions are not evolving free of constraint.

## Introduction

Thirty years ago, King and Wilson [1] raised a key question in human evolutionary genetics: Given that humans and chimpanzees have extremely similar genomes, what can account for the large biological differences between the two species? They proposed the provocative hypothesis that changes in the regulation of gene expression have played a central role in defining these differences.

Since then, some progress has been made toward understanding the genetic basis of human–chimpanzee differences. Most studies have focused on protein coding regions [2–10]. In comparison, much less progress has been made toward understanding the functional significance of noncoding sequence evolution, and as a result, it has been hard to assess King and Wilson's hypothesis.

An important step in assessing that hypothesis is to examine the level of selective constraint in hominid noncoding regions. The approach taken in a recent study was to divide noncoding sequences upstream of genes into 500-bp blocks [11]. Divergence in these blocks was then compared with divergence in putatively neutral regions. This analysis suggested that hominid noncoding regions are essentially evolving free of selective constraint.

Here we take a different approach. Functional elements in noncoding regions can be quite small. As a result, significant variation in hominid divergence may occur on relatively small scales, e.g., less than 50 bp. To capture variation in divergence on this fine scale, we use conservation in more distantly related mammals and amniotes to identify small sequence windows that are likely functional. Interspecies comparisons between different orders of mammals and between mammals and other vertebrates have been used for many years to identify potentially functional noncoding sequences [12–14]. Using this method, we find that that human–chimpanzee divergence is highly correlated with the degree of conservation across mammals and amniotes. That is, using conservation in more distantly related species allows us to find regions that are under strong constraint in hominids. Our results argue that hominid noncoding regions are not evolving free of constraint.

## Results/Discussion

We examined alignments of 10-kb upstream noncoding sequence for 5,547 human–chimpanzee orthologous gene pairs [15] (see Materials and Methods). For each pair we also obtained 10 kb of upstream noncoding sequence for the corresponding mouse, dog, and chicken orthologs. We included only genes for which the 10-kb upstream sequences do not contain any other genes as annotated in the Ensembl database [16]. Using these data, we examined, one small sequence window at a time, how the level of nucleotide divergence in the human–chimpanzee alignment was influenced by the degree of conservation among more distantly related species.

We first used human–mouse–dog three-way comparisons to obtain information on mammalian conservation. We used exhaustive ungapped comparisons to obtain a conservation score for every 16-bp window in the human sequence. Scores were integers between 10 and 16, with higher numbers indicating stronger conservation (see Materials and Methods). We then located each human 16-bp window in the human–chimpanzee alignment and examined the single nucleotide positions immediately to its left and right. This allowed us to calculate the level of human–chimpanzee divergence next to windows of a particular conservation score. For example, we took every window in the human–chimpanzee alignment with a score of 12 as defined by the human–mouse–dog three-way comparisons and determined whether nucleotide sites adjacent to it were the same or different between human and chimpanzee. We then divided the number of sites that showed a difference by the total number of sites, which produces a fraction that indicates the level of human–chimpanzee divergence at conservation score 12. We tabulated human–chimpanzee divergence for each of the conservation scores between 10 and 16.

The motivation for examining adjacent sites, rather than sites that are part of the 16-bp window itself, is to avoid ascertainment bias. If we used sites within the 16-bp conservation window to calculate human–chimpanzee divergence, it would cause bias since the same human nucleotides would contribute to both scores. By using adjacent sites, we avoid this problem. For a description of simulations that illustrate that our method is unbiased, see Materials and Methods and Figure S1.

**Figure 1:**
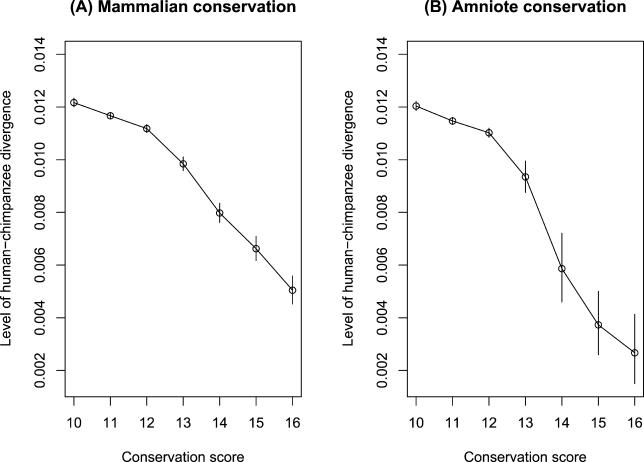
Levels of Human–Chimpanzee Divergence for Different Conservation Scores Conservation scores are calculated using either human–mouse–dog three-way comparisons (A) or human–mouse–chicken comparisons (B). Error bars represent 95% confidence intervals.

Figure 1A shows that broader mammalian conservation is tightly correlated with conservation in hominids (see Table S1 for raw data). Sites next to highly conserved windows are about 40% as likely to have a human–chimpanzee difference as sites next to windows with low scores. That is, mammalian conservation is a good predictor of human–chimpanzee conservation.

We next substituted chicken sequences for dog and repeated the analysis (see Materials and Methods). We found that the conservation score in the human–mouse-chicken comparison is an even better predictor of human–chimpanzee divergence (Figure 1B; also see Table S2 for raw data). These results suggest that many sequences in hominid noncoding regions are highly constrained.

A possible explanation for this pattern is that the upstream regions used in the study contain some surreptitious genes. Our 10-kb sequences do not contain any genes as annotated in the Ensembl database [16]. However, it is possible there are some genes present that are not annotated in Ensembl. To address this possibility, we used transcript predictions from ab initio prediction programs [17,18] in order to eliminate all upstream sequences that contained predicted transcripts (see Materials and Methods). These programs have a relatively liberal definition of genes [19], which allowed us to be more stringent in identifying sequences that do not contain genes in them. Our more stringent set included 2,390 genes. The relationship between human–chimpanzee divergence and conservation score for this set was not appreciably different from that for the whole data set (Figure S2A).

Another possible explanation is that errors in identifying transcription start sites might have somehow contributed to our results. We therefore divided the 10-kb upstream region into two equal halves and repeated the analysis with each. We found that the 5′ and 3′ halves did not differ significantly (Figure S2B). In addition, we tried restricting our analysis to 627 genes whose transcription start site is annotated in the Vertebrate Genome Annotation (Vega) database [20], which is manually annotated. The results for this subset did not differ from those for the whole set of genes (Figure S2C). This indicates that our results are unlikely due to errors in assigning transcription start sites.

We also repeated our analysis by examining only non-CpG sites (i.e., sites that did not overlap a CG dinucleotide in chimpanzee or human), and the pattern remained the same (Table S3).

**Figure 2:**
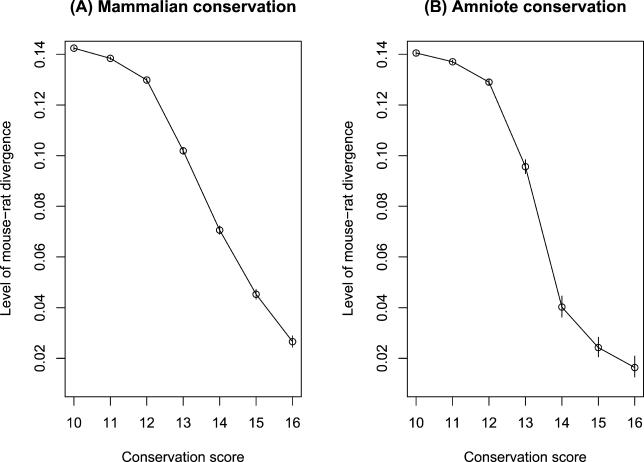
Levels of Mouse–Rat Divergence for Different Conservation Scores Conservation scores are calculated using human–mouse–dog three-way comparisons (A) or human–mouse–chicken comparisons (B). Error bars represent 95% confidence intervals.

For comparison, we repeated our analysis with mouse–rat alignments (Figure 2; also see Tables S4 and S5 for raw data). An obvious difference with Figure 1 is that divergence values are much higher for the murids, reflecting the greater time since their last common ancestor and the faster substitution rate in these lineages. As in the hominids, divergence declines with increasing conservation score. However, in murids the rate of this decline is about twice as high as in hominids, which is similar to that observed by others [21]. This is true even at the most highly conserved sites (e.g., near windows that are the same in human, mouse, and chicken). There are a number of possible explanations for this difference, including sequencing errors in the chimpanzee, relaxation of constraint, positive selection, or the more recent common ancestry of hominids. Errors in the draft chimpanzee genome sequence are likely to be random relative to conservation score and would therefore tend to bring the human–chimpanzee plot closer to random expectation (i.e., make the plot more shallow). Using computer simulations, we found that an error rate of roughly 3 × 10^−3^ would be sufficient to account for the different relative rates of decline in hominids and murids (data not shown). The error rate in the chimpanzee genome sequence is thought to be an order of magnitude less than this and therefore is not enough to account for the hominid–murid differences [15]. Based on polymorphism data from human populations, relaxation of constraint seems preferable to positive selection [11,15,21]. However, there is one other explanation to consider. Because of the recent common ancestry of hominids, approximately 14% of the single nucleotide differences between the human and chimpanzee genomes are at sites polymorphic in one or both species, a value that is likely to be substantially greater than the number for murids [15]. Such sites include some mildly deleterious mutations that have not yet been selected out of the population. This would tend to make the hominid plot shallower than the murid plot. The relative contribution of this factor versus other factors such as relaxation of constraint and positive selection can be better accessed as we get a better appreciation on the nature of sequence polymorphisms in human populations. Also, as more mammalian species are sequenced, we will have other examples of closely relates species that can be compared to hominids.

We also sought to confirm our results using a second, alignment-based method. We downloaded multiple alignments to 1-kb upstream noncoding regions of human genes from the University of California Santa Cruz (UCSC) Genome Informatics Web site (http://hgdownload.cse.ucsc.edu/goldenPath/hg17/multiz8way/). We then examined human–chimpanzee divergence in these relative to the alignments of mouse, dog, and chicken. Table 1 shows our results. We divided nucleotide sites in the human–chimpanzee alignment into several categories: (1) sites where mouse or dog do not align, (2) sites where they do but have differing bases, (3) sites where mouse and dog align and are the same, and (4) sites where mouse, dog, and chicken all align and are the same. Human–chimpanzee divergence decreases as we move from category 1 to category 4. This is also true if we only examine non-CpG sites (Table S6). This is consistent with the results of our previous method, suggesting that sites under constraint among mammals or amniotes generally also tend to be under constraint in hominids.

**Table 1:**
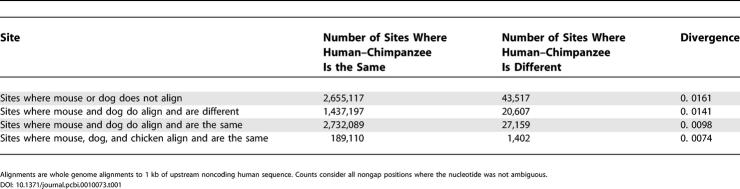
Human–Chimpanzee Divergence Relative to Mouse, Dog, and Chicken Alignments

The simplest explanation of our results is that purifying selection is at work in hominid noncoding sequences. A possible alternative, however, is that those sites which are conserved in amniotes tend to have a low mutation rate and that this low mutation rate alone explains the lower human–chimpanzee divergence. To test this, we made use of the fact that mutation rate variation occurs on a relatively large spatial scale of tens of kilobases [22]. In contrast, purifying selection can act on a much finer spatial scale. In the results given in Table 1, we took a position in the multiple alignment, examined the mouse and dog bases at that site, and then looked at the human–chimpanzee bases at the same site. To test the mutation rate hypothesis, we modified this procedure and instead of looking at the corresponding site for human and chimpanzee, we looked at sites 1 or 15 bases away. If the mutation rate explanation is correct, then the divergence at sites close by should not differ sharply from the rate at the site corresponding to the mouse–dog position. As Table 2 shows, however, there is in fact a substantial difference as we move away in the alignment. The state of mouse and dog at a given alignment position has much more predictive power for the corresponding position in chimp and human than it does for positions 1 or 15 bp away. Again, the same is true if we restrict the analysis to non-CpG sites (Table S7). These results argue that our earlier observations are due to purifying selection rather than low mutation rate.

**Table 2:**
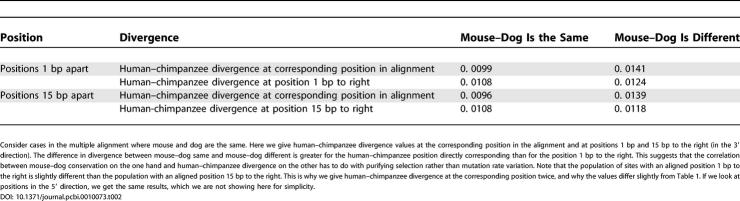
Human–Chimpanzee Divergence at Adjacent Sites in Multiple Alignments with Mouse and Dog

**Figure 3:**
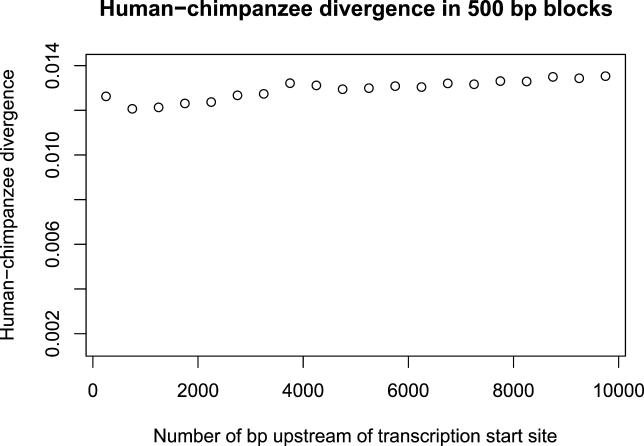
Human–Chimpanzee Divergence in 500-bp Blocks over Our 10-kb Upstream Noncoding Sequences Y axis range is the same as in Figure 1.

Our findings differ from the results of Keightley et al. [11]. Their analysis suggested that hominid noncoding regions have been evolving under exceptionally weak, if any, selective constraint. In contrast, we find evidence that many small sequence windows have evolved under strong constraint. Keightley and colleagues' analysis involved dividing upstream regions into 500-bp blocks. In Figure 3, we present a plot of a similar analysis done with our data, which clearly shows that the range of divergence is much smaller. This likely results from the fact that in large 500-bp blocks, functional elements that are under constraint are mixed with large sections of nonfunctional DNA, which are not under constraint. Because of this, we think that our method of using small sequence windows is a more sensitive way to detect constraint in hominid noncoding regions.

In our data, the difference between murids and hominids is present but is much smaller than that suggested by Keightley and colleagues. We conclude that hominid noncoding regions are subject to significant amounts of selective constraint, though the magnitude of such constraint may not be equal to that observed in other lineages such as murids.

## Materials and Methods

### Acquisition of sequences and alignments.

We obtained a list of human–mouse-dog orthologs via Ensmart (http://www.ensembl.org) and selected the trios that were unique reciprocal best hits [16]. We then used the Ensembl Perl api to identify genes among these whose 5′ upstream regions do not contain another Ensembl gene within 10 kb. For each ortholog trio for which this was true in all three species, we downloaded 10 kb of upstream sequence from the human [23,24], mouse [25], and dog (The Broad Institute, Cambridge, Massachusetts, United States, and Agencourt Bioscience, Beverly, Massachusetts, United States) genomes via Ensembl. All sequences were premasked for repetitive sequence using Repeat Masker (http://www.repeatmasker.org). For the same set of genes, we also obtained a copy of the UCSC human–chimpanzee and mouse–rat blastz alignments via Ensembl's perl api. There were 5,547 ortholog trios for which we obtained a human–chimpanzee alignment and 5,434 trios for which we obtained a mouse–rat alignment. We also repeated this process to get a set of human–mouse–chicken orthologs and downloaded the corresponding chicken genome sequence from Ensembl [26]. There were 3,223 human–mouse–chicken ortholog trios with a human–chimpanzee alignment.

### Calculating conservation scores.

To calculate conservation scores for human noncoding sequences, we used human–mouse–dog three-way comparisons for mammalian conservation and, separately, human–mouse–chicken three-way comparisons for amniote conservation. We chose to use exhaustive ungapped comparison methods [27–29], which have been shown to be highly effective in finding *cis*-regulatory elements [30–34]. Such methods were particularly attractive here because of their simplicity, lack of assumptions, and suitability for producing a distribution of scores. In particular, this approach makes no assumption about the size, amount of similarity, or relative positions of functional elements in the noncoding sequence of various species [29]. For these reasons, the method can detect conserved elements even if their positions have been scrambled during evolution, as long as these elements still lie in the general vicinity of the gene.

We implemented the method using the python interface to the open source Paircomp library developed by Brown et al. [29]. We made use of several functions in that library to calculate a maximum transitive threshold (MTT) conservation score (see Figure S3 for an illustration). Take the example of a 16-bp window from a 10-kb sequence upstream to a human gene. To obtain the MTT conservation score of this window in mammals, we compared it against sequences upstream to the mouse and dog orthologs (10 kb each). We first compared it against all possible 16-bp windows (and their reverse complements) in these two species. For each comparison we obtained a score. In our data, scores are integers between 10 and 16, with higher scores indicating more similarity (e.g., 16 for perfect identity, 15 if there was one mismatch, and so on). Windows with scores under 10 were lumped with score 10 windows. We then considered every combination of three windows where one window comes from each species. For each combination we look at the three pairwise comparisons between species and take the minimum similarity score (e.g., if human–mouse and human–dog are 12, but dog–mouse is 11, then we take 11). We then find the combination of three windows that has the largest minimum similarity score. This score is the MTT, which is a measure of mammalian conservation for the human window. Another way to say this is: we identify the maximum threshold we can set where the given human window has hits in both mouse and dog, and where the mouse and dog hits also hit each other above threshold. Figure S4 shows a plot of these scores, giving the probability of various scores as a function of position relative to the gene.

For comparing 10-kb sequences across distantly related species, previous studies have found a window size of 20 to be suitable [30–34]. We chose to use a slightly smaller window size of 16. This choice represents a tradeoff between two considerations: (1) smaller windows are sensitive to smaller features in the sequence and (2) smaller windows increase the probability of obtaining high scores by random chance. Simulations showed that under our parameters (10-kb sequences and 16-bp windows), high MTT scores are highly unlikely by chance. In a simulation of over 5,000 repetitions where random sequences were compared, there were no windows with an MTT of 15 or 16, and the bulk of scores were 10 or 11 (Table S8). We also tried performing our analysis with nonorthologous human, mouse, and dog upstream sequences (Table S9). The results show that there are essentially no high scoring windows compared with data from orthologous upstream sequences (see Table S1). This shows that our MTT scores reflect conservation rather than random or nonorthologous similarity.

### Correlating human–chimpanzee divergence with conservation score.

For every 16-bp window in the human sequence, we calculated a conservation score as described above. We then located this window in the human–chimpanzee alignment and determined whether the sites immediately to the left and right (not overlapping with the window) were the same or different between human and chimpanzee (Figure S5). We omitted windows that had repetitive sequences in them. For each conservation score, we tabulated the total number of adjacent sites that were the same or different and calculated human–chimpanzee divergence as the number of sites that differed between the two species divided by the total number of sites compared.

### Analysis and simulations.

The 95% confidence intervals for the level of divergence were calculated by bootstrapping over genes 10,000 times. We performed several simulations to check our method for bias and help interpret the results.

We set up a random mutation simulation, which showed that our method is unbiased. We started with our sets of mouse–dog–human orthologs, taking 10 kb of upstream sequence from each. We then took the human sequence, and applied random mutations to it to create two new “species.” We used a Jukes-Cantor substitution model, applying enough substitutions on average so that our new species would differ by about as much as chimpanzee and human. We then treated these two new species the same way we treated human–chimpanzee in the real analysis. We took one of the two new species, species A, and calculated conservation between it and mouse and dog. Then we examined the alignment between species A and species B. Just as we did with the real data, we tabulated divergence scores at sites adjacent to 16-bp conservation windows. The results for 5,547 ortholog sets are shown in Figure S1. Unlike the plots in Figure 1, this plot is flat. That is, divergence is no different at sites next to windows with a 16 conservation score than it is at sites next to windows with a 10 score. This shows that our method is not biased.

We also performed a simulation to assess the potential effect of errors in the chimpanzee genome sequence. We again made two new “species” by applying mutations to human sequences. But this time, we applied them nonrandomly, taking mammalian conservation into account in determining the probability of a mutation at a given nucleotide. We then applied random errors to one of the species (in analogy to sequencing errors in the chimpanzee). By applying different amounts of these random errors, we explored the potential effect of different levels of sequencing errors.

### Analysis of multiple alignments.

Multiple alignments of chimpanzee, mouse, dog, rat, chicken, zebrafish, and fugu to 1 kb of human upstream sequence were downloaded from the UCSC Genome Informatics Web site. For consistency with our initial analysis, we used only mouse, dog, chimp, and chicken. The genes in this data set all have annotated 5′ UTRs. We used the UCSC table browser [35] to identify the subset of these upstream sequences that do not overlap with genes in the UCSC Known Genes track. We then divided the alignment positions into the categories given in Table 1 and calculated human chimpanzee divergence at each. In order to examine whether mutation rate variation might explain our results, we modified the above analysis, this time examining human–chimpanzee sites 1 or 15 bp away. We repeated this analysis with non-CpG sites by eliminating all sites that overlapped a CG dinucleotide in either human or chimpanzee. Analysis was done with a combination of perl and python scripts.

## Supporting Information

Figure S1 Results of a Simulation Illustrating Our Method Applied to Truly Random Substitutions Unlike the plots in Figure 1, this plot is flat, i.e., divergence values next to windows with a 16 score are not different from those next to windows with 10 scores. This shows that our method is not biased (see Materials and Methods for details of the simulation).
(21 KB PDF)

Figure S2 Plots of Proportions As in Figure 1
(A) Data for our full set of 5,547 genes plotted along with those for a stringent “no gene” set of 2,390 genes. For this set we used more stringent criteria in eliminating upstream sequence that might contain a gene.
(B) We divided our 10-kb sequence in half. Here we plot data for the 5′ and 3′ regions separately.
(C) Data for our full set of genes plotted along with a subset of 627 that were manually annotated in Vega.
(29 KB PDF)

Figure S3 Calculating an MTT for the Window on Top (e.g., from Human) against Two Longer Sequences (e.g., from Mouse and Dog) Consider all possible combinations of three 16-bp windows where one window comes from each species. Here we highlight two such combinations in red and blue. For each combination we consider the three pairwise comparisons and take the minimum similarity score. For the window combinations indicated in red, this is 12, and for the combinations indicated in blue, it is 10. We then find the combination (or combinations) of three windows that has the largest minimum similarity score. This score is the MTT. Here the MTT for the window on top is 12. Note that we also consider all the combinations of reverse complements.
(23 KB PDF)

Figure S4 Plots of the Probability of MTT Scores 14, 15, and 16 as a Function of Position Upstream of the Transcription Start Site This is for human–mouse–dog three-way comparisons. In this plot, the probability is averaged over 50-bp regions. Conservation increases significantly near to the gene; 26.7% of all 16 scores, 23.9% of all 15 scores, and 19.7% of all 14 scores occur within 500 bp of the transcription start site.
(56 KB PDF)

Figure S5 A Section of Aligned Sequence (Made Up for Illustrative Purposes) We have already taken human upstream sequence and calculated MTT conservation scores for every 16-bp window. We now take all windows with a particular score and find them in the alignment. Imagine for example that the three windows we have highlighted in blue represent all the windows with a 13 score. We examine the positions adjacent to these, here highlighted in red, and count the number of nucleotides that are the same or different. For our windows with a 13 score, four are the same and two are different. We repeat this for the other possible window scores, creating a table such as Table S1.
(21 KB PDF)

Table S1 Human–Chimpanzee Differences Relative to Mammalian Conservation (17 KB PDF)

Table S2 Human–Chimpanzee Differences Relative to Amniote Conservation (17 KB PDF)

Table S3 Non-CpG Version of Table S1: Human–Chimpanzee Differences Relative to Mammalian Conservation (17 KB PDF)

Table S4 Mouse–Rat Differences Relative to Mammalian Conservation (17 KB PDF)

Table S5 Mouse–Rat Differences Relative to Amniote Conservation (17 KB PDF)

Table S6 Non-CpG Version of Table 1
(26 KB PDF)

Table S7 Non-CpG Version of Table 2
(27 KB PDF)

Table S8 MTT Scores Based on Random Sequence (17 KB PDF)

Table S9 MTT Scores from 10 kb of Upstream Human Noncoding Sequence Compared to Nonorthologous 10-kb Upstream Sequences from Mouse and Dog (17 KB PDF)

## Abbreviations

MTT: maximum transitive threshold
UCSC: University of California Santa Cruz
