# Supplementary material for: Selective Constraint on Noncoding Regions of Hominid Genomes
Source: PLoS Comput Biol. 2005 Dec 16;1(7):e73. doi: 10.1371/journal.pcbi.0010073 (PMC1314883; doi:10.1371/journal.pcbi.0010073)
Supplement: Supplementary file 1 [file pcbi.0010073.sg001.pdf]

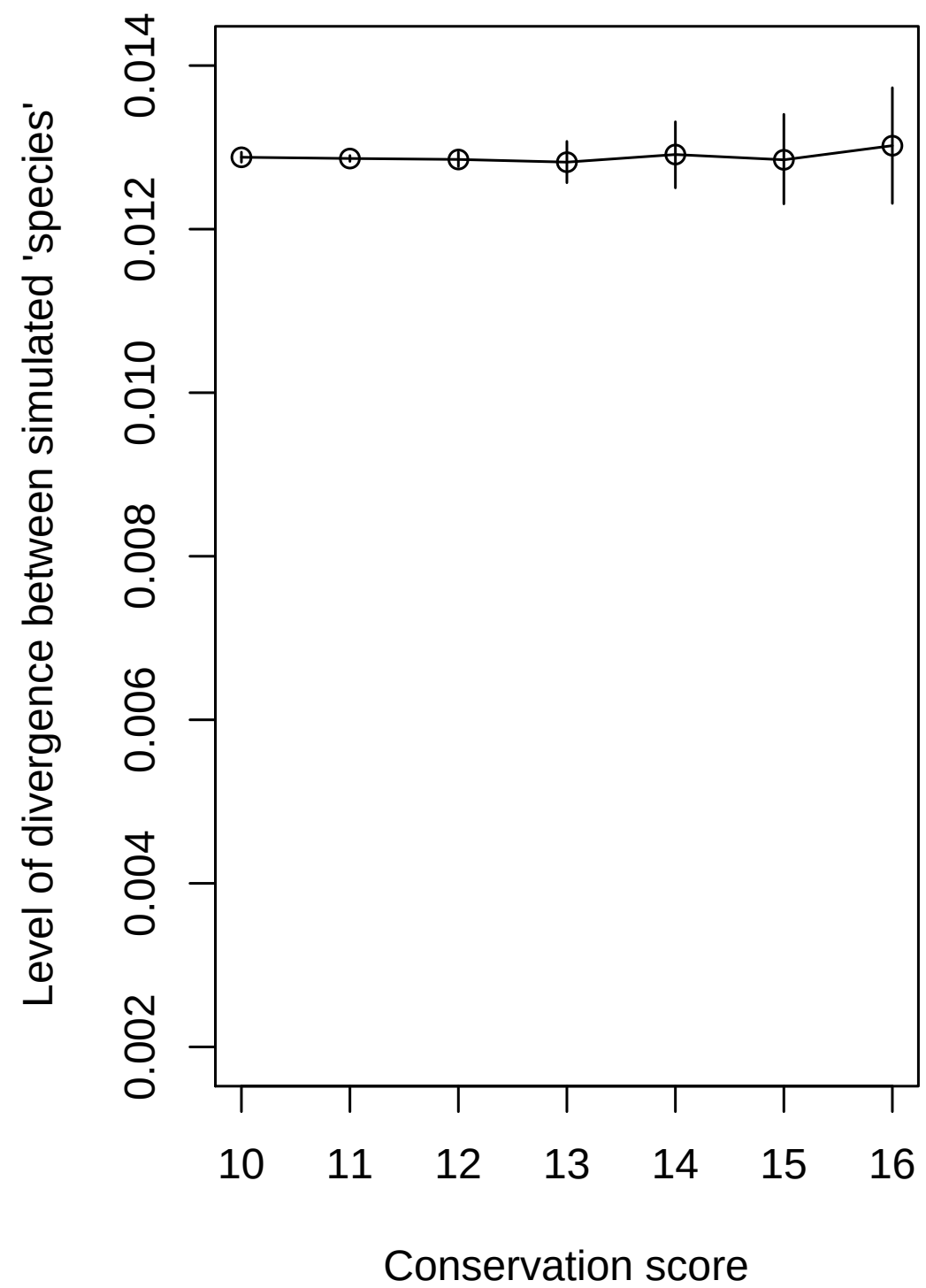

.

Supplementary Figure S1: Results of a simulation illustrating our method applied to truly random substitutions. Unlike the plots in fig. 1, this plot is flat, ie divergence values next to windows with a 16 score are not different from those next to windows with 10 scores. This shows that our method is not biased. (see Methods for details of the simulation).
