# Supplementary material for: Selective Constraint on Noncoding Regions of Hominid Genomes
Source: PLoS Comput Biol. 2005 Dec 16;1(7):e73. doi: 10.1371/journal.pcbi.0010073 (PMC1314883; doi:10.1371/journal.pcbi.0010073)
Supplement: Supplementary file 2 [file pcbi.0010073.sg002.pdf]

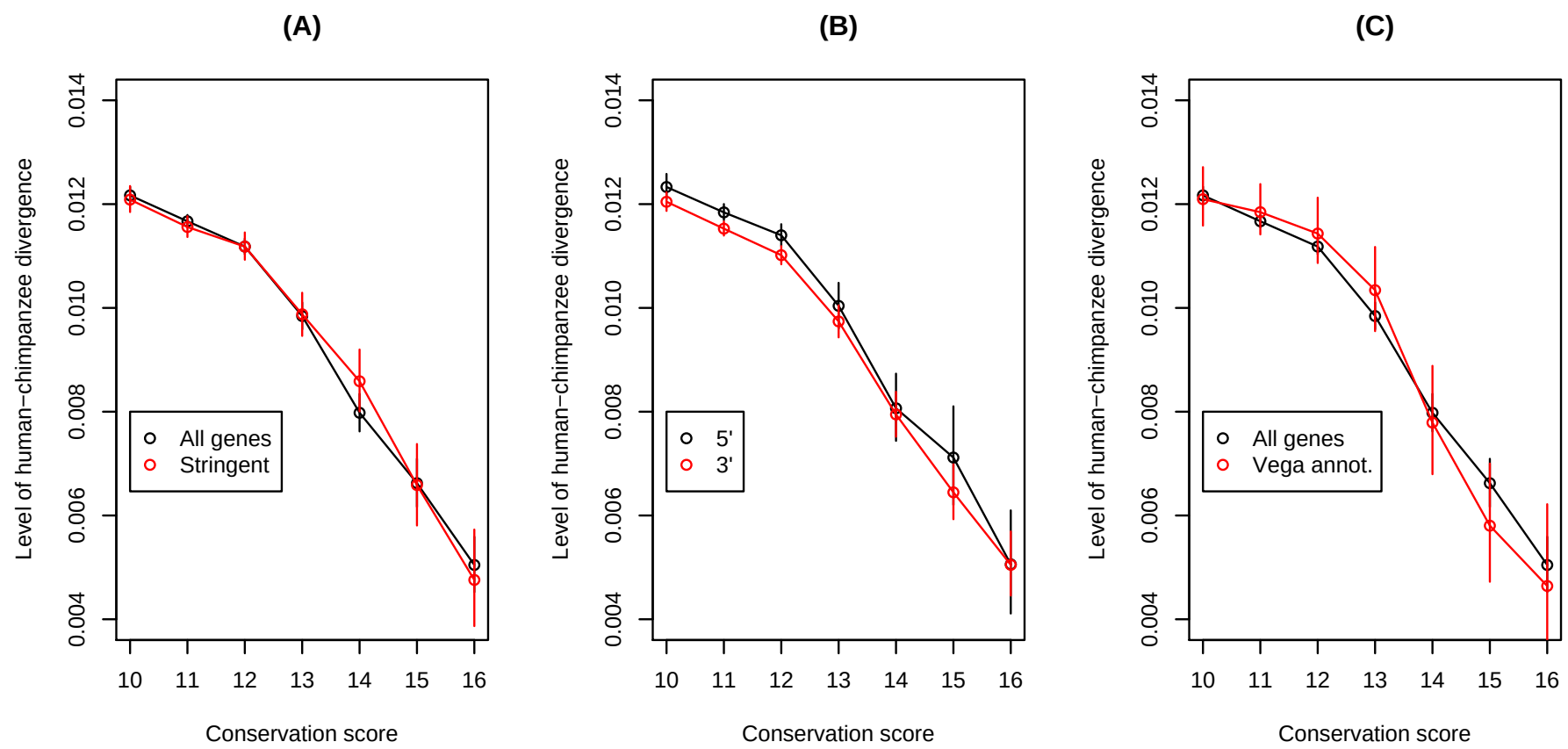

Supplementary Figure S 2: Plots of proportions as in fig. 1. (A) Data for our full set of 5,547 genes plotted along with those for a stringent 'no gene' set of 2,390 genes. For this set we used more stringent criteria in eliminating upstream sequence which might have a gene in it. (B) We divided our 10kb sequence in half. Here we plot data for the 5´ and 3´ regions separately. (C) Data for our full set of genes plotted along with a subset of 627 which were manually annotated in Vega.
