# Supplementary material for: Selective Constraint on Noncoding Regions of Hominid Genomes
Source: PLoS Comput Biol. 2005 Dec 16;1(7):e73. doi: 10.1371/journal.pcbi.0010073 (PMC1314883; doi:10.1371/journal.pcbi.0010073)
Supplement: Supplementary file 3 [file pcbi.0010073.sg003.pdf]

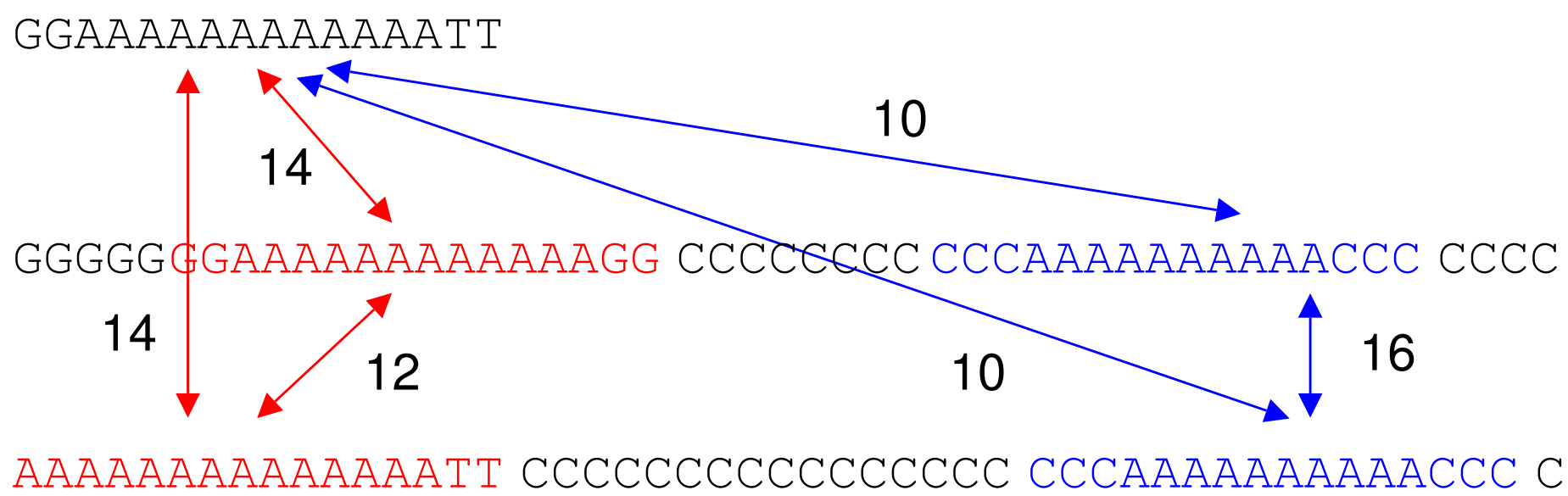

Supplementary Figure S3: Calculating a maximum transitive threshold (MTT) for the window on top (e.g. from human) against two longer sequences (e.g. from mouse and dog). Consider all possible combinations of three 16 bp windows where one window comes from each species. Here we highlight two such combinations in red and blue. For each combination we consider the 3 pairwise comparisons and take the minimum similarity score. For the window combinations indicated in red this is 12, and for the combinations indicated in blue it is 10. We then find the combination (or combinations) of 3 windows which has the largest minimum similarity score. This score is the MTT. Here the MTT for the window on top is 12. Note that we also consider all the combinations of reverse complements.
