# Supplementary material for: Selective Constraint on Noncoding Regions of Hominid Genomes
Source: PLoS Comput Biol. 2005 Dec 16;1(7):e73. doi: 10.1371/journal.pcbi.0010073 (PMC1314883; doi:10.1371/journal.pcbi.0010073)
Supplement: Supplementary file 4 [file pcbi.0010073.sg004.pdf]

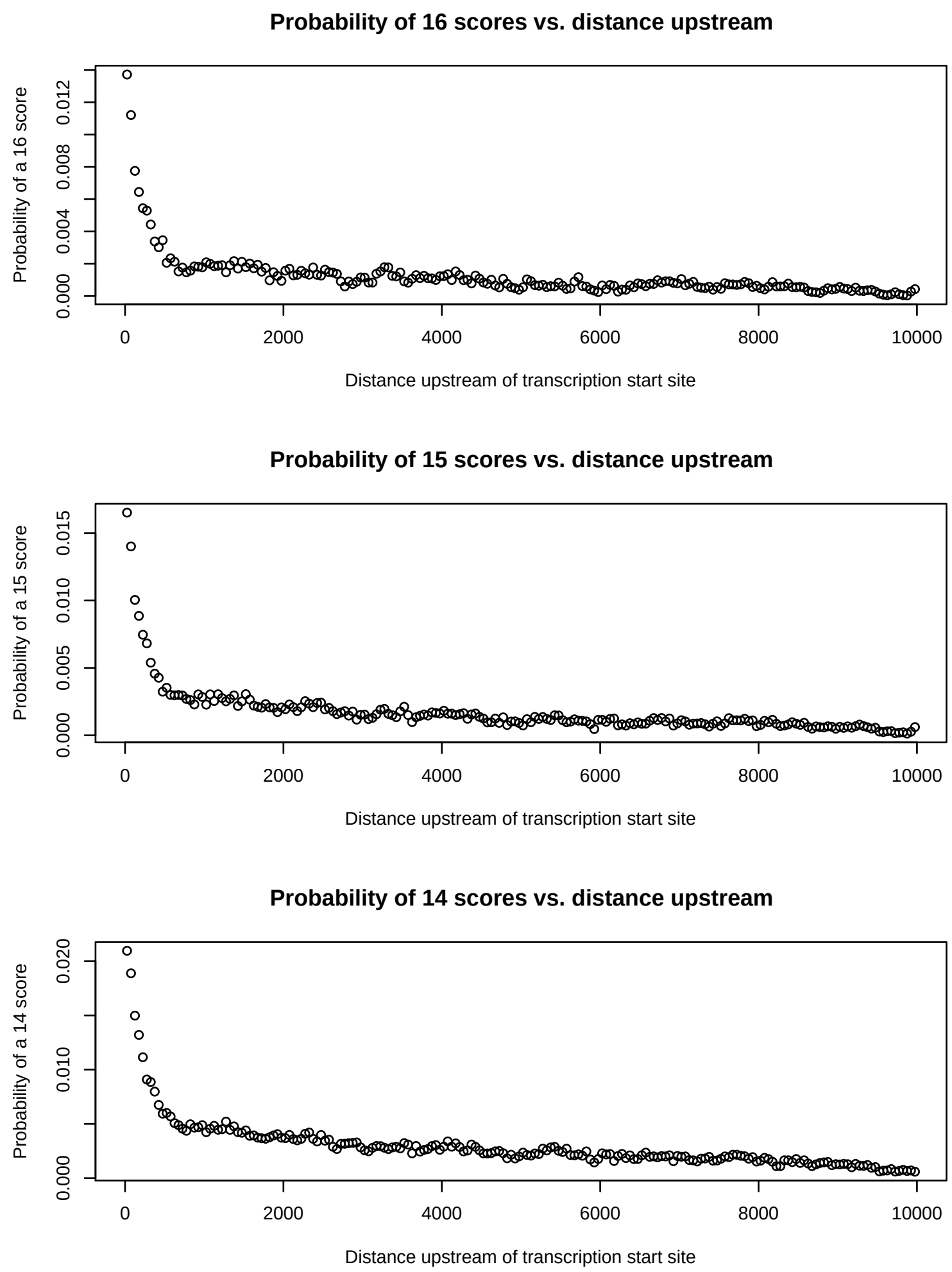

Supplementary Figure S4: Plots of the probability of MTT scores 14, 15, and 16 as a function of position upstream of the transcription start cite. This is for human, mouse, dog three way comparisons. In this plot, the probability is averaged over 50 bp regions. Conservation increases significantly near to the gene. 26.7% of all 16 scores, 23.9% of all 15 scores, and 19.7% of all 14 scores occur within 500 bp of the transcription start site.
