# Supplementary material for: Selective Constraint on Noncoding Regions of Hominid Genomes
Source: PLoS Comput Biol. 2005 Dec 16;1(7):e73. doi: 10.1371/journal.pcbi.0010073 (PMC1314883; doi:10.1371/journal.pcbi.0010073)
Supplement: Supplementary file 5 [file pcbi.0010073.sg005.pdf]

```
pt  AAGTCAGGTCCTCTCGAGATCCTAAGTCCTGCAATACCGCGTATTGCATGATCTAACCCTGGGTACATTACAAAGTGAAGTC
hs  AAGTCAGGTCCTCTCGAGATCCTAAGTCCTG-AATACCGCGTATTGCATGATCTAGCCCTGGGTACATTACAAAGTCAAGTC
```

Supplementary Figure S 5: A section of aligned sequence (which we made up for illustrative purposes). We have already taken human upstream sequence and calculated MTT conservation scores for every 16 bp window. We now take all windows with a particular score and find them in the alignment. Imagine for example that the three windows we have highlighted in blue represent all the windows with a 13 score. We examine the positions adjacent to these, here highlighted in red, and count the number of nucleotides which are the same or different. For our windows with a 13 score, four are the same and two are different. We repeat this for the other possible window scores, creating a table such as supplementary table 1.
