# Supplementary material for: Selective Constraint on Noncoding Regions of Hominid Genomes
Source: PLoS Comput Biol. 2005 Dec 16;1(7):e73. doi: 10.1371/journal.pcbi.0010073 (PMC1314883; doi:10.1371/journal.pcbi.0010073)
Supplement: Supplementary file 6 [file pcbi.0010073.st001.pdf]

Conservation score	Same	Different	Proportion different
10	12,692,801	156,349	0.01217
11	30,226,313	356,888	0.01167
12	5,862,525	66,291	0.01118
13	765,091	7,606	0.00984
14	310,972	2,501	0.00798
15	176,402	1,175	0.00662
16	124,102	629	0.00504

Supplementary Table S1: Human-chimpanzee differences relative to mammalian conservation. Counts for the number of same and different nucleotides in the human-chimpanzee alignment for different conservation window scores. Here conservation scores were calculated using human-mouse-dog three way comparisons. Also given is the proportion different (level of divergence).
