# Supplementary material for: Selective Constraint on Noncoding Regions of Hominid Genomes
Source: PLoS Comput Biol. 2005 Dec 16;1(7):e73. doi: 10.1371/journal.pcbi.0010073 (PMC1314883; doi:10.1371/journal.pcbi.0010073)
Supplement: Supplementary file 7 [file pcbi.0010073.st002.pdf]

Conservation score	Same	Different	Proportion different
10	6,603,542	80,453	0.01204
11	21,146,072	245,351	0.01147
12	3,101,449	34,562	0.01102
13	130,654	1,233	0.00935
14	18,167	107	0.00586
15	10,180	38	0.00372
16	7,950	21	0.00263

Supplementary Table S 2: Human-chimpanzee differences relative to amniote conservation. Counts for the number of same and different nucleotides in the human-chimpanzee alignment for different conservation window scores. Here conservation scores were calculated using human-mouse-chicken three way comparisons. Also given is the proportion different (level of divergence).
