# Supplementary material for: Selective Constraint on Noncoding Regions of Hominid Genomes
Source: PLoS Comput Biol. 2005 Dec 16;1(7):e73. doi: 10.1371/journal.pcbi.0010073 (PMC1314883; doi:10.1371/journal.pcbi.0010073)
Supplement: Supplementary file 8 [file pcbi.0010073.st003.pdf]

Conservation score	Same	Different	Proportion different
10	12,216,333	109,898	0.00892
11	29,420,851	262,900	0.00886
12	5,705,704	49,918	0.00867
13	736,850	5,624	0.00757
14	295,262	1,766	0.00595
15	166,086	783	0.00469
16	116,420	441	0.00377

Supplementary Table S3: Non-CpG version of Supplementary Table S1. Human-chimpanzee differences relative to mammalian conservation. Counts for the number of same and different nucleotides in the human-chimpanzee alignment for different conservation window scores. Here conservation scores were calculated using human-mouse-dog three way comparisons. Also given is the proportion different (level of divergence).
