# Supplementary material for: Selective Constraint on Noncoding Regions of Hominid Genomes
Source: PLoS Comput Biol. 2005 Dec 16;1(7):e73. doi: 10.1371/journal.pcbi.0010073 (PMC1314883; doi:10.1371/journal.pcbi.0010073)
Supplement: Supplementary file 9 [file pcbi.0010073.st004.pdf]

Conservation score	Same	Different	Proportion different
10	13,394,127	2,224,853	0.1424
11	24,538,779	3,942,005	0.1384
12	4,600,929	686,663	0.1299
13	656,521	74,499	0.1019
14	283,908	21,564	0.0706
15	168,516	7,986	0.0452
16	123,092	3,351	0.0265

Supplementary Table S4: Mouse-rat differences relative to mammalian conservation. Counts for the number of same and different nucleotides in the mouse-rat alignment for different conservation window scores. Here conservation scores were calculated using human-mouse-dog three way comparisons. Also given is the proportion different (level of divergence).
