# Supplementary material for: Selective Constraint on Noncoding Regions of Hominid Genomes
Source: PLoS Comput Biol. 2005 Dec 16;1(7):e73. doi: 10.1371/journal.pcbi.0010073 (PMC1314883; doi:10.1371/journal.pcbi.0010073)
Supplement: Supplementary file 10 [file pcbi.0010073.st005.pdf]

Conservation score	Same	Different	Proportion different
10	6,915,434	1130797	0.1405
11	17,238,886	2738356	0.1371
12	2,396,752	355080	0.1290
13	110,496	11684	0.0956
14	17,234	721	0.0402
15	10,153	252	0.0242
16	7,909	130	0.0162

Supplementary Table S 5: Mouse-rat differences relative to amniote conservation. Counts for the number of same and different nucleotides in the mouse-rat alignment for different conservation window scores. Here conservation scores were calculated using human-mouse-chicken three way comparisons. Also given is the proportion different (level of divergence).
