# Supplementary material for: Selective Constraint on Noncoding Regions of Hominid Genomes
Source: PLoS Comput Biol. 2005 Dec 16;1(7):e73. doi: 10.1371/journal.pcbi.0010073 (PMC1314883; doi:10.1371/journal.pcbi.0010073)
Supplement: Supplementary file 11 [file pcbi.0010073.st006.pdf]

	# Sites hs-pt same	# Sites hs-pt Diff	Divergence
Mouse or dog does not align	2,441,719	30,461	0.0123
Mouse and dog do align and are different	1,322,715	13,937	0.0104
Mouse and dog do align and are same	2,547,302	19,336	0.0075
Mouse,dog and chicken align, and are same	172,857	970	0.0056

Supplementary Table S6: Non-CpG version of Table 1. Human-chimpanzee divergence relative to mouse, dog and chicken alignments. Alignments are whole genome alignments to 1kb of upstream human sequence. Counts consider all non-gap positions where the nucleotide was not ambiguous. Here we also eliminated all sites which overlapped with a CG in either human or chimpanzee.
