# Supplementary material for: Selective Constraint on Noncoding Regions of Hominid Genomes
Source: PLoS Comput Biol. 2005 Dec 16;1(7):e73. doi: 10.1371/journal.pcbi.0010073 (PMC1314883; doi:10.1371/journal.pcbi.0010073)
Supplement: Supplementary file 12 [file pcbi.0010073.st007.pdf]

		Mouse-Dog Same	Mouse-Dog Diff
Pos 1 bp apart	Hs-Pt diverg. at corresponding position	0.0075	0.0103
	Hs-Pt diverg. at position 1 bp to right	0.0081	0.0093
Pos 15 bp apart	Hs-Pt diverg. at corresponding position	0.0074	0.0101
	Hs-Pt diverg. at position 15 bp to right	0.0080	0.0089

Supplementary Table S 7: Non-CpG version of Table 2. Consider cases in the multiple alignment where mouse and dog are the same. Here we give human-chimpanzee divergence values at the corresponding position in the alignment, and at positions 1 and 15 bp to the right (in the 3' direction). The difference in divergence between mouse-dog same and mouse-dog different is greater for the human-chimpanzee position directly corresponding than for the position 1 bp to the right. This suggests that the correlation between mouse-dog conservation on the one hand and human-chimpanzee divergence on the other has to do with purifying selection rather than mutation rate variation. Note that the population of sites which have an aligned position 1bp to the right is slightly different than the population which have an aligned position 15 bp to the right. This is why we give human-chimpanzee divergence at the corresponding position twice, and why the values differ slightly from Supplementary table 6. If we look at positions in the 5' direction, we get the same results, which we are not showing here for simplicity.
