# Supplementary material for: Selective Constraint on Noncoding Regions of Hominid Genomes
Source: PLoS Comput Biol. 2005 Dec 16;1(7):e73. doi: 10.1371/journal.pcbi.0010073 (PMC1314883; doi:10.1371/journal.pcbi.0010073)
Supplement: Supplementary file 13 [file pcbi.0010073.st008.pdf]

Conservation score	Count
10	14,365,868
11	39,423,942
12	1,579,723
13	17,133
14	129
15	0
16	0

Supplementary Table S8: MTT scores based on random sequence. We generated sets of three random 10 kb sequences and calculated MTT scores from these. The counts given here come from 5,547 iterations of this. With our parameters (window size = 16 bp, sequence length = 10kb) high window scores are very unlikely by random chance.
