# Supplementary material for: Selective Constraint on Noncoding Regions of Hominid Genomes
Source: PLoS Comput Biol. 2005 Dec 16;1(7):e73. doi: 10.1371/journal.pcbi.0010073 (PMC1314883; doi:10.1371/journal.pcbi.0010073)
Supplement: Supplementary file 14 [file pcbi.0010073.st009.pdf]

Conservation score	Count
10	19,954,761
11	28,421,170
12	2,326,741
13	46,229
14	742
15	2
16	0

Supplementary Table S9: MTT scores from 10kb of upstream human noncoding sequence when compared against NON-ORTHOLOGOUS 10kb upstream sequences from mouse and dog. Data come from 5,547 sets, just as in supplementary table 1. There are very few high scoring windows here. This shows that the high scoring windows in supplementary table 1 are the result of matches between conserved orthologous sequences.
